# Spatial Characterization of the Human Meniscus: Bridging Biomechanics and Biochemistry Using Atomic Force Microscopy Micro‐Indentation and Mass Spectrometry Imaging

**DOI:** 10.1002/smtd.70908

**Published:** 2026-08-11

**Authors:** Aleksandra N. Lebedeva, Martin Handelshauser, Orestis G. Andriotis, Lena Hirtler, Martina Marchetti‐Deschmann, Philipp J. Thurner

**Affiliations:** ^1^ Faculty of Mechanical and Industrial Engineering Institute of Lightweight Design and Structural Biomechanics TU Wien Vienna Austria; ^2^ Center For Anatomy and Cell Biology Medical University of Vienna Vienna Austria; ^3^ Faculty of Technical Chemistry Institute of Chemical Technologies and Analytics TU Wien Vienna Austria

**Keywords:** AFM micro‐indentation, collagen, human meniscus, MALDI mass spectrometry imaging, multimodal imaging

## Abstract

Meniscus biomechanical properties are dependent on the microscale composition and distribution of structural proteins and other macromolecules. In this context, the local mechanical properties (material properties) of the meniscus largely depend on the chemical composition. However, to date, a direct link between the spatial biomechanical and biochemical footprint of the meniscus at the microscale is lacking. To this end, we show a multimodal imaging approach for fresh frozen tissue linking the biomechanical and biochemical readouts from atomic force microscopy (AFM) micro‐indentation and matrix‐assisted laser desorption/ionization mass spectrometry imaging (MALDI MSI), respectively. We consecutively applied both imaging modalities on the same tissue section, enabling direct correlative comparison across nano‐ to micrometer length scales. Using human meniscus cryosections, we provide spatial distribution information of collagen and other proteins in three zones of the meniscus section exhibiting different apparent elasticity. This methodology shows promise in providing a better understanding of the relationship between local meniscus composition and micromechanics. In addition, the technique has potential for improved detection of meniscus degeneration biomarkers and, in the future, linking those to clinical imaging readouts.

## Graphical abstract

A multimodal imaging approach combines AFM micro‐indentation and MALDI mass spectrometry imaging on the same fresh‐frozen human meniscus section to directly associate microscale mechanics with molecular composition. Collagen‐rich regions exhibit increased stiffness, whereas zones enriched in other extracellular matrix proteins are mechanically less stiff, establishing a direct biomechanical—biochemical link across meniscal tissue.

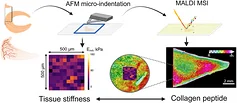

## 1 Introduction

Meniscus degeneration remains one of the leading causes of chronic knee pain in patients [1, 2, 3, 4]. Meniscal injuries, which are among the most common conditions encountered in clinical practice, are also a major risk factor for the development of knee osteoarthritis (OA) [1, 4, 5, 6, 7].

To assess tissue mechanical function and the local mechanical microenvironment experienced by resident cells, atomic force microscopy (AFM)‐based indentation and force‐mapping approaches have emerged as powerful tools for quantitative micro‐ and nanomechanical characterization of biological tissues [8, 9, 10, 11]. Recent advances in AFM nanomechanical mapping have further expanded the ability to investigate heterogeneous biological tissues through quantitative force‐based imaging with improved spatial resolution [9, 10, 11]. In AFM micro‐indentation experiments, a spherical probe is pressed into the tissue surface while force–indentation curves are recorded and analyzed using contact mechanics models to determine apparent mechanical properties [9, 10, 12, 13]. Beyond single‐point measurements, AFM force‐mapping allows spatially resolved characterization of tissue mechanical heterogeneity, providing quantitative information on local stiffness and other nanomechanical parameters [9, 10, 11]. Furthermore, AFM measurements can be performed in liquid environments under near‐physiological conditions, require minimal sample preparation, and are largely non‐destructive, enabling subsequent complementary analyses of the same specimens [10, 11, 14].

Complementary to biomechanical characterization, MALDI MSI has become an important tool in clinical research due to its ability to detect spatially resolved molecular features and potential disease biomarkers [15]. When combined with enzymatic digestion (e.g., Trypsin/Lys‐C), MALDI MSI allows for detailed mapping of proteins like collagens and other extracellular matrix (ECM) components in cartilage tissue, revealing their identity, distribution, capturing both unmodified and post‐translationally modified peptides [16, 17, 18]. Such molecular maps can highlight tissue alteration, degeneration and/or inflammation, aiding the identification of OA‐related biomarkers [19].

Tissue biomechanics and composition of the meniscus are tightly linked as cells respond to mechanical load to influence their mechanical microenvironment [20, 21, 22]. While AFM and MALDI MSI are imaging modalities that can be combined into a multimodal workflow to explore such biochemical links—as has been shown to some extent for inorganic materials [23, 24]—changes in meniscal composition are expected to alter local tissue biomechanics [20, 25]. In this context, the interplay between mechanical properties and molecular composition is highly relevant for meniscus degeneration and the subsequent development of knee OA [25]. To address this, we sequentially applied first AFM micro‐indentation and second MALDI MSI on the same meniscus cryosection. This combined methodology enables a correlative analysis of meniscus micro‐stiffness and biochemical composition, providing unprecedented insights into the structural and molecular characteristics underlying meniscal function.

## 2 Results and Discussion

**FIGURE 1:**
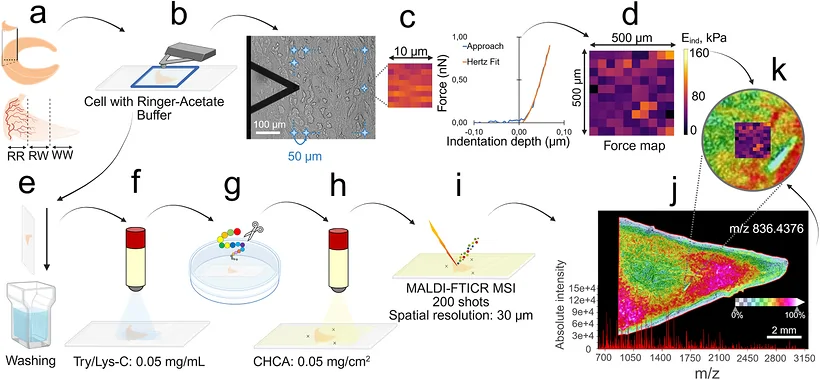
Schematic of the experimental workflow for multimodal imaging of human meniscus sections: (a) Illustration of human meniscus cryosectioning and different zones of a vertical meniscus section (RR—red‐red, RW—red‐white, WW—white‐white) [6]; (b) AFM micro‐indentation of meniscus tissue in liquid; (c) AFM force mapping of meniscus region of interest (ROI) with 50 µm step; single force map (10 × 10 µm, 8×8 pixels) and Hertz fitting of the obtained force‐indentation curve (R^2^ > 0.95); (d) Mean indentation modulus map over a total scanned ROI (500 × 500 µm); Sample preparation for MALDI MSI measurements: (e) section rinsing, (f) Try/Lys‐C spraying on tissue section, (g) incubation, (h) matrix spraying and (i) MALDI MSI; (j) Profile mass spectrum of ROI ion image of COL1α1/ COL2α1/COL3α1 GPPGPQGAR m/z 836.4376 unmodified peptide and representative brightfield image of the meniscus section; (k) Image correlation of AFM and MALDI MS imaging. Created with BioRender.com.

To establish a correlative workflow linking microscale mechanics and molecular composition, three consecutive cryosections of human meniscus tissue were analyzed using a combined AFM–MALDI MSI approach (Figure 1). AFM micro‐indentation was first employed to map regional variations in indentation modulus across distinct meniscal zones. The same tissue sections were subsequently subjected to MALDI‐FTICR mass spectrometry imaging, enabling spatially resolved visualization of extracellular matrix–derived peptides. This sequential analysis allowed direct comparison of biomechanical and biochemical features within the same anatomical context and serves as a proof‐of‐concept for multimodal characterization of human meniscal tissue.

### 2.1 Spatial Distribution of ECM Peptides in the Meniscus Tissue

**FIGURE 2:**
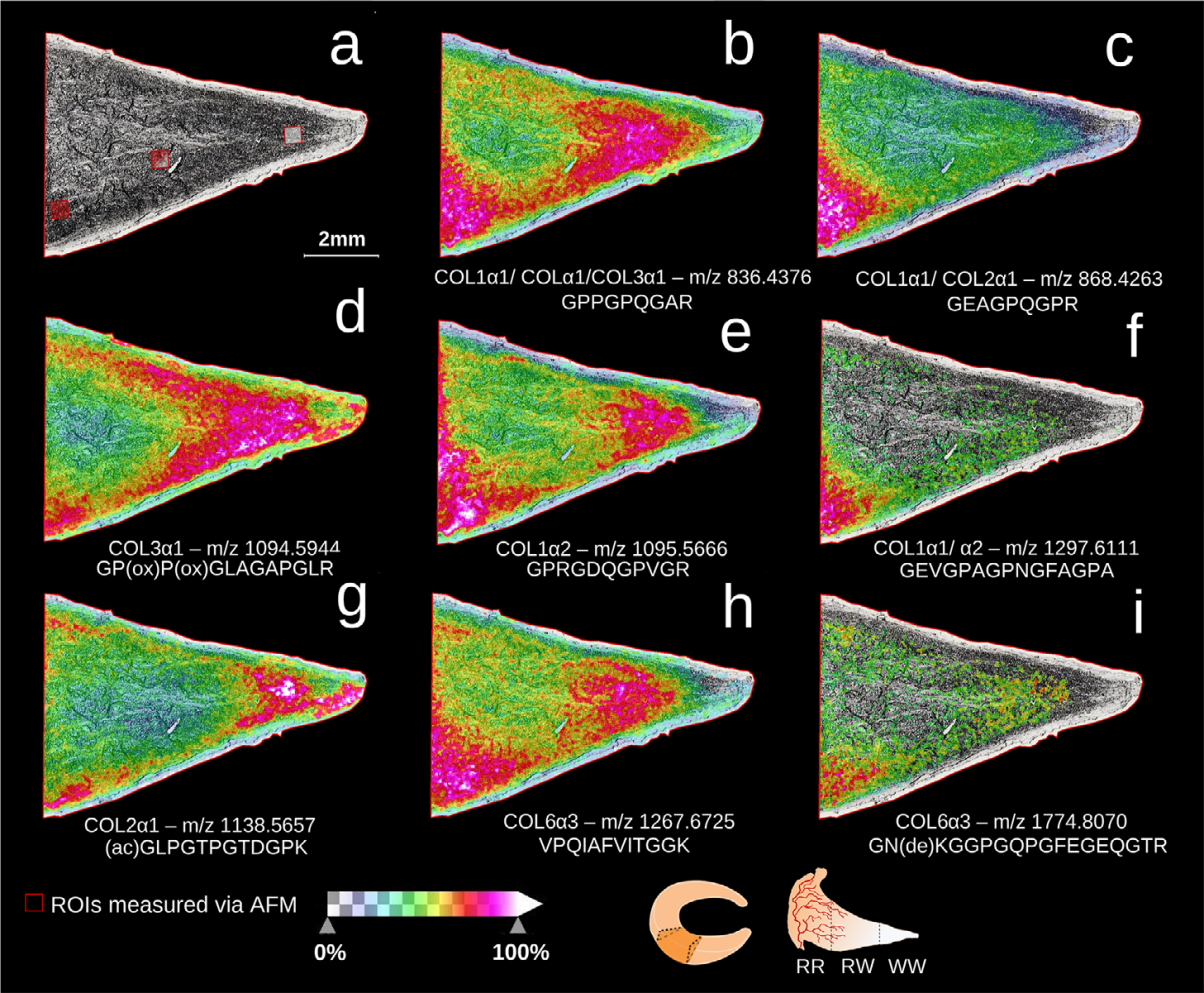
(a) An optical image of a human meniscus section after MALDI MSI analysis with marked AFM indented regions. Ion images with a spatial distribution of TIC‐normalized ion intensities of monoisotopic peptide signals: (b) m/z 836.4376, collagen type I alpha 1 chain/collagen type II alpha 1 chain/collagen type III alpha 1 chain (COL1α1/ COL2α1/COL3α1), unmodified; (c) m/z 868.4263, collagen type I alpha 1 chain/collagen type II alpha 1 chain (COL1α1/ COL2α1), unmodified; (d) m/z 1094.5944, collagen type III alpha 1 chain (COL3α1), 2 P‐oxidation (ox); (e) m/z 1095.5666, collagen type I alpha 2 chain (COL1α2), unmodified; (f) m/z 1297.6111, collagen type I alpha 1 and alpha 2 chains (COL1α1/α2), unmodified; (g) m/z 1138.5657, collagen type II alpha 1 chain (COL2α1), acetylation (ac); (h) m/z 1267.6725, collagen type VI alpha 3 chain (COL6α3), unmodified; (i) m/z 1774.8070, collagen type VI alpha 3 chain (COL6α3), deamidation (de). Scale bar: 2 mm. Color scaling represents signal intensity levels from 0% to 100% for the same threshold level for all images.

Representative mass spectra of human meniscus tissue are shown in Figures 1j, S1,S5 and S12. Ion images of several abundant peptides, normalized to the total ion current (TIC), are presented in Figure 2. The primary structural components of the meniscus are collagen types I and II (COL1 and COL2) [5, 6, 7, 26], and this study focused on the detection and spatial distribution of peptides derived from these two collagen types. COL1 is composed of two α1(I) chains and one α2(I) chain, whereas COL2 is composed of three α1(II) chains [27]. COL1 molecules self‐assemble into larger fibrillar structures, termed collagen fibrils. Collagen fibrils provide tensile strength and resistance to shear forces [27, 28].

In our MALDI‐FTICR MSI dataset, numerous monoisotopic peptide signals matched collagen isoforms with high mass accuracy. COL1 is the dominant collagen molecule in meniscal tissue; unmodified peptide at m/z 1297.6111 COL1α1/α2 and the unique peptide at m/z 1095.5666 for COL1α2 show increased intensity in the lamellar layer of both red‐red (outer) and white‐white (inner) zones (Figure 2e,f). A similar localization pattern was observed for additional peptides associated with COL1, such as m/z 836.4376 and 868.4263 (Figure 2b,c). The peptide at m/z 1297.6111 (COL1α1/α2) was identified as a specific sequence shared between both α‐chains of COL1 (Table S1), further supporting its specificity as a marker for this collagen type [29, 30].

COL2 is the second major component of meniscus tissue and is primarily found in the avascular inner zone (white‐white zone) [5, 6, 25]. COL2 molecules form collagen fibrils, but with reduced diameter compared to COL1 [27, 31]. The unique N‐terminally acetylated peptide GLPGTPGTDGPK of COL2α1 (m/z 1138.5657) shows a lateral distribution resembling that of COL1, with increased signal intensity in the white‐white zone and near the superficial layer.

COL1 typically forms fibrils in association with collagen III (COL3), which plays a role in collagen fibril organization and contributes to tissue elasticity [27, 32, 33]. Notably, we observed particularly strong signal intensity for the oxidized peptide GP(ox)P(ox)GLAGAPGLR (m/z 1094.5944), which is unique for COL3α1, in the white‐white zone. This finding is of interest, because while COL2 has previously been described as the most abundant protein in this zone, COL3 may also play a more significant role in the inner meniscus than previously recognized. Furthermore, a previous study indicated that COL3 often retains its N‐terminal pro‐peptide in cartilage and meniscus, which may influence its fibrillogenesis and matrix interactions [34].

COL1, COL2 and COL3 produce highly similar tryptic peptides because they share the same fundamental triple‐helical structure composed of repeating Gly–X–Y motifs and undergo similar post‐translational modifications [27, 35]. Consequently, peptides derived from these collagens overlap. In all experiments, a common peptide at m/z 836.4376, corresponding to either COL1α1, COL2α1 or COL3α1, was consistently detected (Figure 2b). This shared peptide was localized primarily in the lamellar layer, with enhanced signal in both the white‐white and red‐red zones (Figure 2b). Additional ion images for these collagens from two replicate experiments, as well as corresponding images displayed without transparent overlay, are provided in the Supplementary Information (Figures S2,S6,S7,S13,S14).

Collagen VI (COL6) forms a microfibrillar network within the ECM of the meniscus, where it is enriched in the pericellular space and functions as a structural and signaling bridge between fibrillar collagens (I, II, III) and meniscal fibrochondrocytes [36, 37]. While COL6 does not form fibrils, it co‐localizes with fibrillar collagens in the meniscus because its microfibrillar network anchors to COL1, COL2 and COL3 [27, 38]. This interaction integrates the pericellular matrix with the bulk fibrillar matrix, so regions rich in fibrillar collagens also exhibit strong COL6 signals at m/z 1267.6725 and 1774.8070 (Figure 2h,i). The overlapping distribution observed in our MSI maps therefore reflects the structural role of COL6 as a molecular bridge that stabilizes fibrillar networks and mediates cell–matrix interactions. Immunohistochemical studies in human meniscal tissue confirm the presence of COL6 throughout, including pericellular localization [36, 39]. This spatial pattern reinforces our MSI observations of COL6 co‐distribution with fibrillar collagens in similar anatomical zones.

Several of these assignments for COL1α1 (e.g. m/z 836.4373 (GPAGPQGPR/GPPGPQGAR), m/z 868.4290 (GEAGPQGPR), m/z 886.4388 (GSEGPQGVR), m/z 1297.6093 (GESGPSGPAGPTGAR)) and COL1α2 (e.g., m/z 868.4635, 1235.6126, 2078.9631) have already been previously reported and cross‐validated by LC–MS/MS [29, 30]. Additional assignments for COL3 and COL6 show overlap with other LC–MS/MS‐based proteomic investigations [29], further supporting our peptide identifications (Table S1).

Beyond collagens, the meniscus contains various other ECM components and proteins. Ion images of clusterin (CLU, also known as apolipoprotein J), in its unmodified form (m/z 779.4060) and as a deamidated (NQ) version (m/z 1076.5201) are shown in Figure S3b,c. These signals are localized in the deep layer of the red‐white zone in the central part of the section. CLU is a glycoprotein and potential inflammatory marker that is frequently upregulated in response to mechanical stress [40]. Figure S3d,e display the distribution of prolargin (PRELP), a small leucine‐rich proteoglycan. Signals at m/z 954.5128 and m/z 1070.5965 were enriched in the deep and lamellar layers, occupying regions between the collagen‐rich zones. PRELP is known to bind to COL1 and COL2, anchoring within the ECM and contributing to the regulation of fibril spacing and organization [40]. The unique PRELP peptide ISSVPAINNR (m/z 1070.5965) was also reported in human osteoarthritic cartilage [41]. Figure S3f,g show signals corresponding to vitronectin (VTNC), detected at m/z 1314.6797, and 1422.6502, localized in the superficial layer near the white‐white zone. These two peptides were also reported in amyloidogenic human liver tissue in a previous MSI study [42]; however, for m/z 1314.6797 we observed a different sequence (INCQGKTYLFK). Although VTNC is not a primary structural component, matrix glycoprotein VTNC participates in cell adhesion, fibrinolysis, and tissue remodeling [43, 44]. In Figure S3g–i, we also observed enhanced intensities of PGS2 peptides at m/z 1176.5440 and 1325.5835 in the inner zone. PGS2 is again a small proteoglycan involved in collagen fibrillogenesis, specifically regulating fibril diameter and spacing [45].

### 2.2 Image Segmentation and Zonal Biomechanical Characteristics

**FIGURE 3:**
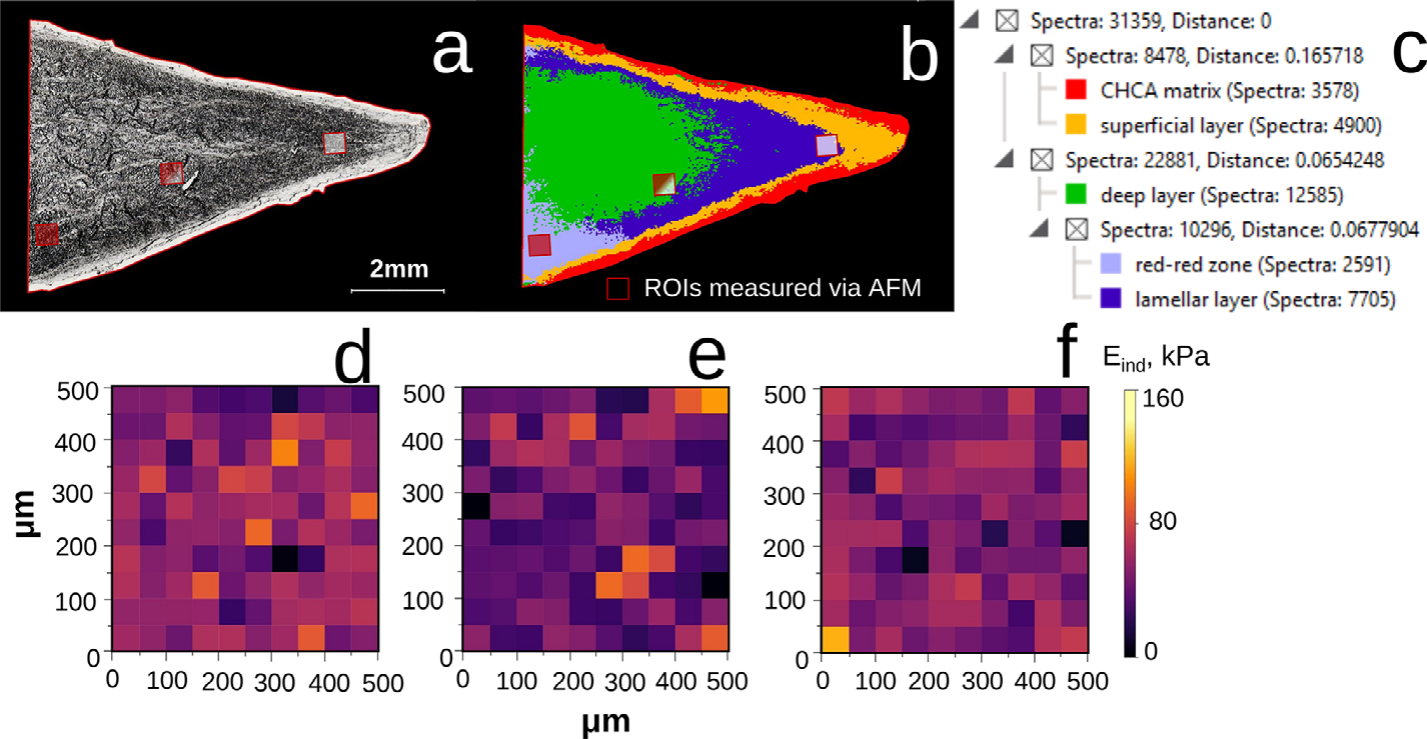
(a) An optical image of a human meniscus section after MALDI‐FTICR MSI analysis with marked AFM indented regions; (b) MALDI‐FTICR MSI segmentation (bisecting *k*‐means clustering) of a human meniscus section; (c) Corresponding dendrogram illustrating the hierarchical relationship of identified clusters. Indentation modulus maps of the marked AFM indented regions in: (d) red‐red (outer) zone, (e) red‐white (middle) zone, (f) white‐white (inner) zone. Created with BioRender.com.

Image segmentation based on regional, mass spectral similarities using bisecting *k*‐means clustering distinguished four major tissue compartments within the human meniscus section (Figure 3b). These included the superficial layer at the outer surface (orange color), a lamellar layer beneath (dark blue color), a deep layer enriched in glycoprotein‐related signals (green color), and the vascularized red‐red zone at the periphery (light blue color). The segmentation map thus recapitulated the known anatomical zones of the meniscus corroborating further the morphological relevance of the MALDI‐FTICR MSI results. AFM micro‐indentation maps acquired from three representative ROIs (Figure 3d–f) revealed pronounced microscale heterogeneity in stiffness across these regions, with indentation moduli ranging from 10 to 160 kPa. Notably, higher apparent stiffness was observed in lamellar and red‐red zones, where fibrillar collagen peptides (Figure 2) were most abundant in the MSI data, whereas the deep layer exhibited lower stiffness and reduced fibrillar collagen signals. Together, these results highlight that biochemical segmentation based on peptide distributions closely matches biomechanical zonation, reinforcing the link between local ECM composition and microscale mechanical properties [46, 47].

Our indentation moduli (10–160 kPa) overlap with values reported for juvenile bovine meniscus at the nano‐to‐microscale [48], but are lower than those reported for porcine [46] and human intact tissues [47], which often exceed 200 kPa. The discrepancy in absolute values can be explained by methodological differences (probe size, section thickness, orientation effects, indentation rate, indentation depth, Poisson's ratio assumptions) and donor variation (elderly, degenerated human tissue in our study versus younger or animal tissue). Importantly, all studies converge on the same zonal trend, with the outer meniscus stiffer than the inner zone. Our multimodal AFM–MSI data further provide a molecular basis for these differences, demonstrating that stiff regions coincide with fibrillar collagen–rich domains, while softer regions are enriched in non‐fibrillar ECM proteins [49].

**FIGURE 4:**
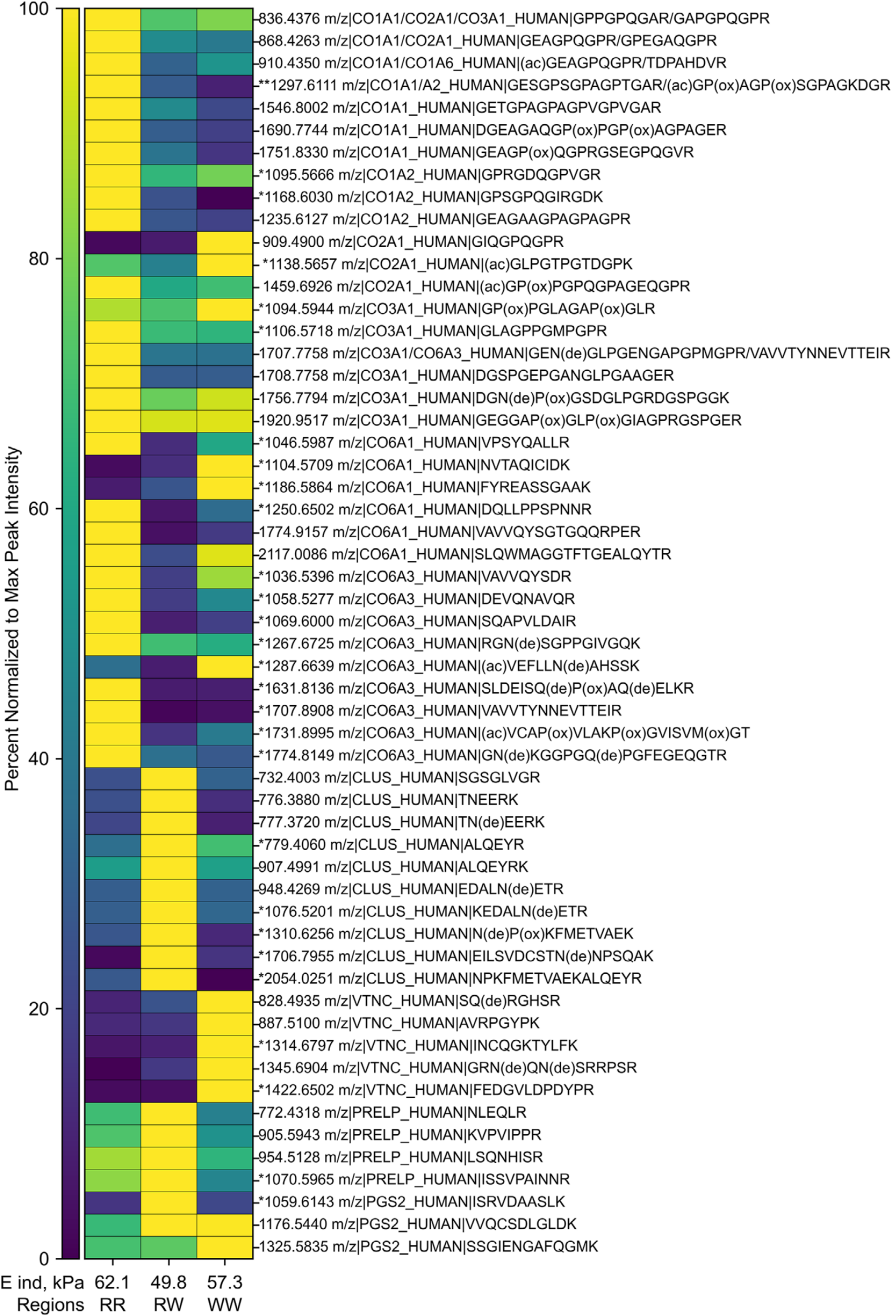
Heatmap of TIC‐normalized peptide ion intensities normalized per m/z across the three meniscal zones (RR, RW, WW), with the maximum regional mean intensity for each m/z set to 100%. Mean indentation modulus values are shown. *Unique peptides; **m/z 1297.6111–collagen I peptide (α1/α2).

The normalized peptide intensity heatmaps (Figure 4; Figures S11 and S18) display TIC‐normalized peptide ion signals across the red‐red, red‐white, and white‐white zones. Each row represents one annotated peptide feature (m/z and protein assignment). Signals were normalized per m/z across regions (maximum = 100%) to emphasize relative spatial patterns rather than absolute intensity differences between peptides. COL6 peptides, along with VTNC, show a marked increase in intensity in the stiffer tissue regions (white‐white and red‐red zones). In contrast, COL1, COL2 and COL3 peptides display relatively uniform intensities across zones, consistent with their roles as structural fibrillar collagens broadly distributed throughout the meniscus. In comparison, CLU, PRELP and PGS2 exhibit higher intensity in the red‐white (softer) zone. The enrichment of small leucine‐rich proteoglycans (SLRPs), such as PRELP and PGS2, reflects active ECM remodeling and altered collagen organization, consistent with biochemical adaptations of less load‐bearing tissue zones [45, 50, 51, 52]. In the prior study, CLU was mostly abundant in the inner and middle zones, the small leucine‐rich proteoglycan PRELP displayed the highest abundance in the red‐white and red‐red zones and the PGS2 had high abundance values in all zones [51]. In our case, the signals for CLU and PRELP are enhanced in the middle region, and PGS2 is present in all zones (Figures S3,S4,S8,S9,S15,S16).

Nesbitt et al. recently reported data from a study on human menisci correlating bulk ECM composition and collagen crosslinks with tensile toughness of mm‐sized specimens [53]. In contrast, the presented AFM/MALDI‐FTICR MSI maps relate local peptide distributions to local microscale indentation modulus. Nesbitt et al. observed age‐related increases in fibrillar collagens and decreases in basement membrane collagens and proteoglycans, suggesting matrix remodeling influences meniscal toughness [53]. Although the mechanical readouts between Nesbitt et al. and our study differ (tensile toughness vs. indentation modulus), both datasets converge on a structure—function relationship wherein collagen network quality and pericellular organization govern performance. Consistently, our stiff regions co‐localize with fibrillar collagens, whereas fewer stiff regions show higher signals from non‐fibrillar ECM such as PRELP, and the reported age‐related loss of COL6 and deoxypyridinoline (DPD), a mature enzymatic collagen crosslink indicative of collagen network stabilization, provides a biochemical rationale for reduced local stiffness and toughness in elderly tissues.

Here, we showcase a multimodal approach combining spatially resolved AFM micro‐indentation for state‐of‐the‐art micromechanical assessment and MALDI‐FTICR MSI, for microscale compositional characterization on the same sample. This gives insight into the spatial distribution of ECM peptides and their relationship to local micromechanical properties of the human meniscus. Nevertheless, some methodological limitations should be acknowledged.

In this presented study, peptide distributions and stiffness values were compared on a regional basis rather than through true pixel‐level co‐registration, limiting the spatial precision of molecular–mechanical correlations. AFM micro‐indentation was carried out on selected 500 × 500 µm ROIs chosen to represent characteristic microenvironments, which does not yet capture the full heterogeneity of entire sections. Future serial sectioning will enable more comprehensive sampling. Peptide assignments were based on mass matching (<10 ppm) supported by Mascot, ExPASy, BLAST, and literature data. Assignments with mass errors ≤3 ppm were classified as high‐confidence annotations, whereas assignments with mass errors between 3 and 10 ppm were considered tentative and are indicated accordingly in the supplementary tables (Table S1). Additional orthogonal confirmation using on‐tissue LC–MS/MS could further strengthen selected peptide annotations, particularly those associated with higher mass deviations. Finally, the dataset comprises only elderly donors, and spatial concordance between AFM and MSI maps was assessed visually. As such, the observed biochemical—mechanical relationships are descriptive and provide a foundation for more detailed, statistically resolved studies. Together, these points highlight several opportunities for future studies, including expanded donor cohorts, improved spatial co‐registration strategies, and additional orthogonal validation of selected peptide assignments.

## 3 Conclusion

In summary, our study presents the combination of AFM and MALDI MSI in tissue analysis to directly link microscale biomechanical and biochemical analyses for the same transverse section of a human meniscus. We successfully acquired force volume maps from three anatomical zones (red‐red, red‐white and white‐white) and applied MSI to the same sections to assess the respective molecular composition. AFM data revealed region‐specific differences in tissue elasticity, while the MSI data showed distinct peptide distributions within the AFM‐scanned regions. The spatially resolved biochemical analysis helps explain the difference in the apparent micro‐stiffness across the three regions. The observed spatial co‐localization of mechanical and molecular features highlights the potential of this multimodal approach for investigating how biomechanical properties relate to the localization of specific ECM proteins in connective tissues.

## 4 Experimental Section/Methods

### 4.1 Reagents and Materials

All reagents were obtained from Sigma‐Aldrich (St. Louis, MO, US) at ≥99% purity. Ultra‐high‐quality water (ddH_2_O; resistivity < 18.2 MΩ·cm at 25°C) was produced using a Simplicity system (Millipore, Billerica, MA, US). Conductive indium tin oxide (ITO)–coated microscope slides were purchased from Delta Technologies (Loveland, MN, US) and were coated with poly‐L‐lysine as follows: a 1:1 mixture (750 µL + 750 µL; total 1.5 mL) of poly‐L‐lysine (0.1% w/v in H_2_O) and MS‐grade water was prepared in a 2 mL microcentrifuge tube. IGEPAL CO‐630 (1 µL) was added and the solution mixed thoroughly. Twenty microliters of the resulting solution were spread homogeneously over the conductive surface of each ITO slide using a clean coverslip as a spreader. Slides were dried in a laboratory oven at 80°C for 15 min. Pre‐coated slides were stored dry at ambient conditions in standard polypropylene slide holders.

### 4.2 Human Tissues

Human meniscus samples originated from voluntary body donations to the Division of Anatomy of the Center for Anatomy and Cell Biology of the Medical University of Vienna. The body donors bequeathed their bodies for use for educational and scientific purposes through written informed consent prior to their death. The study was approved by the ethics committee of the Medical University of Vienna (#1723/2018). Tissue handling and use complied with institutional and national ethical regulations for research involving human specimens. The age range for donors was 70–99 years. Samples were obtained dry and frozen at −80°C. For subsequent cryo‐sectioning, the first step involved embedding meniscus samples. Tragacanth powder was dissolved in Millipore water to produce a jelly‐like medium, which was then shaped into a pyramid within a plastic mold (Figure S19a). The meniscus was dissected into two pieces, using a sterile scalpel. A small cavity was created at the center of the tragacanth pyramid, into which the sample was carefully inserted with tweezers, ensuring that approximately two‐thirds of the tissue protruded above the surface (Figure S19b). The embedded sample was wrapped in aluminum foil and placed in a −70°C laboratory freezer for a minimum of 30 min to ensure complete solidification of the tragacanth embedding medium. Cryo‐sectioning was performed using a CryoStar NX50 cryostat. Before sectioning, the frozen sample was equilibrated at room temperature for 10 min and unwrapped. The sample was mounted on a moistened specimen fixation platform and allowed to adhere inside the cryostat chamber for at least 30 min. Central and peripheral parts of menisci were cut into 10 µm thick transverse cryosections at −20°C and deposited on adhesive poly‐L‐lysine‐coated Indium Tin Oxide slides via thermal contact and dried in a desiccator for at least 1 h. As a proof‐of‐concept demonstration, three consecutive cryosections (technical replicates) obtained from a single human meniscus donor were used. Therefore, the statistical analyses presented in this study are intended to describe regional trends within the investigated specimen rather than biological variability across donors.

### 4.3 AFM Micro‐Indentation

Three consecutive cryosections from a single human meniscus donor (*n* = 1) were investigated via AFM micro‐indentation (Nanowizard Ultraspeed A, JPK‐Bruker) using a cantilever (NP‐O10‐D, 0.06 N/m spring constant and 18 kHz resonance frequency) equipped with a 10 µm diameter borosilicate glass spherical tip (1.0 nN setpoint) [54]. A 3D‐printed frame was glued with silicone around the tissue section for measurements in physiological conditions. The space inside the frame was filled with 1 mL phosphate‐buffered saline (PBS) with a broad‐spectrum protease inhibitor (Ringer acetate buffer pH = 7.4 / Roche cOmplete, Mini, EDTA‐free Protease Inhibitor Tablets, 1 tablet per 10 mL). In each section, three regions of interest (ROIs) were indented in red‐red (outer), red‐white (middle), and white‐white (inner) zones, respectively. Each ROI consisted of force maps (10 × 10 µm, 8 × 8 pixels, pixel size = 1.25 µm) acquired at 50 µm intervals covering a total area of 500 × 500 µm. Force curves were analyzed with the Hertz model [55] using a custom MATLAB program (https://github.com/Rufman91/ForceMapAnalysis) [56]. The mean indentation modulus of each 50 µm position was calculated from the average of all pixels within the corresponding force map. Considering curves with a Hertz fit coefficient of determination > 0.95, the indentation modulus was calculated [55]. For each force map, the mean indentation modulus was calculated from all valid force curves and compared among the RR, RW, and WW regions using the Kruskal–Wallis test followed by Dunn's post hoc multiple‐comparison test, with *p* < 0.05 considered statistically significant (Figure S22).

After AFM micro‐indentation experiments, samples were stored in the freezer at −20°C for up to 2 weeks. Before on‐tissue digestion, samples were dried in a desiccator at least 1 h, lipids and salts were removed by sequential washing in precooled on ice staining jars with 70% ethanol (30 s), 100% ethanol (30 s), Carnoy's solution (120 s), 70% ethanol (30 s), water (30 s) and 100% ethanol (30 s). Between each washing step, samples were dried with nitrogen, after the final step they were dried in desiccator for at least 15 min. Samples were sprayed with 0.05 mg/mL Trypsin/Lys‐C in ammonium hydrogen carbonate (pH = 8.0) by HTX TM‐Sprayer (HTX Technologies LLC, 45°C, 10 psi, flow rate 0.5 mL/min, 8 passes, loop volume 5 mL) and incubated for 3 h at 37°C in a home‐built humidification chamber. After incubation, 10 mg/ml α‐cyano‐4‐hydroxy‐cinnamic acid (CHCA) in a solution of 0.1% trifluoracetic acid (TFA) and acetonitrile (ACN) (2:3 vol) was also applied via spraying (60°C, 10 psi, flow rate 0.15 mL/min, 8 passes, velocity 1200 mm/min, pattern CC, gas flow rate 3 L/min, drying time 30 s, nozzle height 40 mm, loop volume 5 mL). The amount of matrix deposited per unit of tissue surface is 0.5 mg/cm^2^. Samples were measured on a 7T MALDI‐FTICR system (scimaX, Bruker) at 30 µm lateral resolution (200 shots, medium laser diameter 20 µm) between m/z 700 and 3500.

### 4.4 MS Image Generation and Data Analysis

All ion images with the distribution of peptides were co‐registered by SCiLS Lab (Bruker v. 2025a) and normalized to Total Ion Current (TIC). The spectral data were processed using the Bisecting *k*‐means clustering algorithm with weak denoising applied. Peak area was selected as the interval processing mode. Clustering was performed using the correlation distance metric. The analysis was carried out in all‐spectra mode, ensuring comprehensive coverage of the dataset.

Monoisotopic m/z lists were generated from TIC‐normalized data using SCiLS Lab (Bruker v. 2025a) based on feature co‐localization within *k*‐means segments. For protein identification, these lists were submitted to the in‐house Mascot server. To comply with Mascot's m/z limit, approximately 3% of the entire m/z list was excluded prior to the first submission. The search was performed using the following search parameters: enzyme Trypsin, 2 missed cleavages, 10 ppm m/z error, and Acetyl (N‐term), Deamidated (NQ), Oxidation (M), Oxidation (P) as variable modifications. Data were searched against the Uniprot Database “Human collagen‐containing extracellular matrix” v. 27.11.2024, restricted to entries with protein existence (PE) = 1 (evidence at the protein level). Unmatched m/z values from the first search were then combined with the excluded 3% of signals and resubmitted for a second search against the same database. Proteins were considered to be identified if identifications were above a certain threshold level (38, *p* < 0.05). Results are summarized in Table S1.

The regions of interest (ROIs) measured via AFM were imported into SCiLS Lab software as areas of approximately 500 × 500 µm. These three ROIs correspond to the same areas highlighted in red in the optical images (Figure 2a, 3a; Figures S2a–S4a,S6a–S10a,S13a–S17a). For each ROI, average intensity values were extracted for each m/z. For visualization, intensities were normalized per m/z across the compared ROIs in meniscal zones (red‐red/red‐white/white‐white) by dividing each regional mean intensity by the maximum mean intensity among ROIs for that m/z (maximum set to 100%), and expressing the remaining values as percentages of this reference. The heatmaps were generated with custom‐written Python scripts.

### 4.5 Protein IDs

For protein identification, ID criteria included that at least three m/z values had to be assigned to one protein to consider its presence. Additionally, all peaks associated with a protein had to exhibit the same spatial distribution. Mascot searches were performed using a precursor mass tolerance of 10 ppm. For final annotation confidence assessment, peptide assignments with mass errors ≤3 ppm were classified as high‐confidence annotations, whereas assignments with mass errors between 3 and 10 ppm were considered tentative and are indicated accordingly in Table S1. Each selected peak was additionally required to show at least the first isotopic signal. Peptide lists for proteins identified via Mascot search were generated through in silico digestion using Expasy (https://web.expasy.org/peptide_mass/). The settings for this digestion included the following: [M + H]^+^ ions, trypsin was chosen as enzyme, up to two missed cleavages were acceptable, and only peptides between m/z 750 and 3000 were considered. Peptide sequences assigned to certain m/z values were checked by Protein BLAST (https://blast.ncbi.nlm.nih.gov/) for uniqueness against the Uniprot Database for Human. The peptide is considered unique only if multiple alignments did not show further protein options.

## Conflicts of Interest

The authors declare no conflicts of interest.

## Supporting information




**Supporting File 1**: smtd70908‐sup‐0001‐SuppMat.docx.

**Supporting File 2**: smtd70908‐sup‐0002‐Table S1.xlsx.

## Data Availability

The data that support the findings of this study are available from the corresponding author upon reasonable request.

## References

[1] M. Hurmuz , M. Ionac , B. Hogea , C. A. Miu , and F. Tatu , “Osteoarthritis Development Following Meniscectomy vs. Meniscal Repair for Posterior Medial Meniscus Injuries: A Systematic Review,” Medicina (Lithuania) 60, no. 4 (2024): 569, 10.3390/medicina60040569. PMC11052089 38674215

[2] R. L. Mauck and J. A. Burdick , “From Repair to Regeneration: Biomaterials to Reprogram the Meniscus Wound Microenvironment,” Annals of Biomedical Engineering 43 (2015): 529–542, 10.1007/s10439-015-1249-z. PMC4380775 25650096

[3] N. Ozeki , H. Koga , and I. Sekiya , “Degenerative Meniscus in Knee Osteoarthritis: From Pathology to Treatment,” Life 12, no. 4 (2022): 603, 10.3390/life12040603. PMC9032096 35455094

[4] E. Luvsannyam , M. S. Jain , A. R. Leitao , N. Maikawa , and A. E. Leitao , “Meniscus Tear: Pathology, Incidence, and Management,” Cureus 14, no. 5 (2022): 25121, 10.7759/cureus.25121. PMC9205760 35733484

[5] A. J. S. Fox , A. Bedi , and S. A. Rodeo , “The Basic Science of Human Knee Menisci: Structure, Composition, and Function,” Sports Health 4 (2012): 340–351. 10.1177/1941738111429419 PMC3435920 23016106

[6] E. A. Makris , P. Hadidi , and K. A. Athanasiou , “The Knee Meniscus: Structure–Function, Pathophysiology, Current Repair Techniques, and Prospects for Regeneration,” Biomaterials 32 (2011): 7411–7431, 10.1016/j.biomaterials.2011.06.037. PMC3161498 21764438

[7] E. S. Mameri , S. P. Dasari , L. M. Fortier , et al., “Review of Meniscus Anatomy and Biomechanics,” Current Reviews in Musculoskeletal Medicine 15 (2022): 323–335, 10.1007/s12178-022-09768-1. PMC9463428 35947336

[8] M. Stolz , R. Gottardi , R. Raiteri , et al., “Early Detection of Aging Cartilage and Osteoarthritis in Mice and Patient Samples Using Atomic Force Microscopy,” Nature Nanotechnology 4 (2009): 186–192, 10.1038/nnano.2008.410. 19265849

[9] R. Garcia and J. R. Tejedor , “Advances in Nanomechanical Property Mapping by Atomic Force Microscopy,” Nanoscale Advances 7 (2025): 6286–6307, 10.1039/D5NA00702J. PMC12379807 40880595

[10] D. H. Cho , S. Aguayo , and A. X. Cartagena‐Rivera , “Atomic Force Microscopy‐Mediated Mechanobiological Profiling of Complex Human Tissues,” Biomaterials 303 (2023): 122389, 10.1016/j.biomaterials.2023.122389. PMC10842832 37988897

[11] S. Batasheva , S. Kotova , A. Frolova , and R. Fakhrullin , “Atomic Force Microscopy for Characterization of Decellularized Extracellular Matrix (dECM) Based Materials,” Science and Technology of Advanced Materials 25 (2024): 2421739, 10.1080/14686996.2024.2421739. PMC11573343 39559530

[12] F. J. Giessibl , “Advances in Atomic Force Microscopy,” Reviews of Modern Physics 75 (2003): 949–983, 10.1103/RevModPhys.75.949.

[13] M. Loparic , D. Wirz , A. U. Daniels , et al., “Micro‐ and Nanomechanical Analysis of Articular Cartilage by Indentation‐Type Atomic Force Microscopy: Validation With a Gel‐Microfiber Composite,” Biophysical Journal 98 (2010): 2731–2740, 10.1016/j.bpj.2010.02.013. PMC2877335 20513418

[14] J. A. Last , P. Russell , P. F. Nealey , and C. J. Murphy , “The Applications of Atomic Force Microscopy to Vision Science,” Investigative Opthalmology & Visual Science 51 (2010): 6083–6094, 10.1167/iovs.10-5470. PMC3055745 21123767

[15] M. Aichler and A. Walch , “MALDI Imaging Mass Spectrometry: Current Frontiers and Perspectives in Pathology Research and Practice,” Laboratory Investigation 95 (2015): 422–431, 10.1038/labinvest.2014.156. 25621874

[16] M. R. Groseclose , M. Andersson , W. M. Hardesty , and R. M. Caprioli , “Identification of Proteins Directly From Tissue: In Situ Tryptic Digestions Coupled With Imaging Mass Spectrometry,” Journal of Mass Spectrometry 42 (2007): 254–262, 10.1002/jms.1177. 17230433

[17] P. M. Angel , S. Comte‐Walters , L. E. Ball , et al., “Mapping Extracellular Matrix Proteins in Formalin‐Fixed, Paraffin‐Embedded Tissues by MALDI Imaging Mass Spectrometry,” Journal of Proteome Research 17, no. 1 (2017): 635–646, 10.1021/acs.jproteome.7b00713. PMC6637938 29161047

[18] A. M. Judd , D. B. Gutierrez , J. L. Moore , et al., “A Recommended and Verified Procedure for in Situ Tryptic Digestion of Formalin‐Fixed Paraffin‐Embedded Tissues for Analysis by Matrix‐Assisted Laser Desorption/Ionization Imaging Mass Spectrometry,” Journal of Mass Spectrometry 54 (2019): 716–727, 10.1002/jms.4384. PMC6711785 31254303

[19] Y. R. Lee , M. T. Briggs , M. R. Condina , et al., “Mass Spectrometry Imaging as a Potential Tool to Investigate Human Osteoarthritis at the Tissue Level,” International Journal of Molecular Sciences 21 (2020): 1–13. 10.3390/ijms21176414 PMC7503948 32899238

[20] P. X. Bradley , K. N. Thomas , A. L. Kratzer , et al., “The Interplay of Biomechanical and Biological Changes Following Meniscus Injury,” Current Rheumatology Reports 25 (2023): 35–46, 10.1007/s11926-022-01093-3. PMC10267895 36479669

[21] A. L. McNulty and F. Guilak , “Mechanobiology of the Meniscus,” Journal of Biomechanics 48 (2015): 1469–1478, 10.1016/j.jbiomech.2015.02.008. PMC4442061 25731738

[22] M. J. Vyhlidal and A. B. Adesida , “Mechanotransduction in Meniscus Fibrochondrocytes: What About Caveolae?,” Journal of Cellular Physiology 237 (2022): 1171–1181, 10.1002/jcp.30616. 34676536

[23] R. Thiruvallur Eachambadi , H. T. S. Boschker , A. Franquet , et al., “Enhanced Laterally Resolved ToF‐SIMS and AFM Imaging of the Electrically Conductive Structures in Cable Bacteria,” Analytical Chemistry 93 (2021): 7226–7234, 10.1021/acs.analchem.1c00298. 33939426

[24] O. S. Ovchinnikova , K. Kjoller , G. B. Hurst , D. A. Pelletier , and G. J. Van Berkel , “Atomic Force Microscope Controlled Topographical Imaging and Proximal Probe Thermal Desorption/Ionization Mass Spectrometry Imaging,” Analytical Chemistry 86 (2014): 1083–1090, 10.1021/ac4026576. 24377265

[25] D. Warnecke , J. Balko , J. Haas , et al., “Degeneration Alters the Biomechanical Properties and Structural Composition of Lateral Human Menisci,” Osteoarthritis and Cartilage 28 (2020): 1482–1491, 10.1016/j.joca.2020.07.004. 32739340

[26] D. R. Eyre and J. J. Wu , “Collagen of Fibrocartilage: A Distinctive Molecular Phenotype in Bovine Meniscus,” FEBS Letters 158 (1983): 265–270, 10.1016/0014-5793(83)80592-4. 6688225

[27] S. Ricard‐Blum , “The Collagen Family,” Cold Spring Harbor Perspectives in Biology 3 (2011): a004978–a004978, 10.1101/cshperspect.a004978. PMC3003457 21421911

[28] A. Rezazadeh Nochehdehi and F. Nemavhola , Thomas in Cartilage Tissue and Knee Joint Biomechanics: Fundamentals, Characterization and Modelling (Elsevier, 2023), 61–73.

[29] T. S. Høiem , M. K. Andersen , M. Martin‐Lorenzo , et al., “An Optimized MALDI MSI Protocol for Spatial Detection of Tryptic Peptides in Fresh Frozen Prostate Tissue,” Proteomics 22, no. 10 (2022): 2100223, 10.1002/pmic.202100223. PMC9285595 35170848

[30] B. Heijs , S. Holst , I. H. Briaire‐De Bruijn , et al., “Multimodal Mass Spectrometry Imaging of N ‐Glycans and Proteins From the Same Tissue Section,” Analytical Chemistry 88 (2016): 7745–7753, 10.1021/acs.analchem.6b01739. 27373711

[31] M. R. Buckley , E. B. Evans , P. E. Matuszewski , et al., “Distributions of Types I, II and III Collagen by Region in the Human Supraspinatus Tendon,” Connective Tissue Research 54 (2013): 374–379, 10.3109/03008207.2013.847096. PMC6056177 24088220

[32] X. Liu , H. Wu , M. Byrne , S. Krane , and R. Jaenisch , “Type III Collagen is Crucial for Collagen I Fibrillogenesis and for Normal Cardiovascular Development,” Proceedings of the National Academy of Sciences 94 (1997): 1852–1856, 10.1073/pnas.94.5.1852. PMC20006 9050868

[33] J. ChandraRajan , “Separation of Type III Collagen From Type I Collagen and Pepsin by Differential Denaturation and Renaturation,” Biochemical and Biophysical Research Communications 83 (1978): 180–186, 10.1016/0006-291X(78)90414-X. 358975

[34] C. Wang , B. K. Brisson , M. Terajima , et al., “Type III Collagen is a Key Regulator of the Collagen Fibrillar Structure and Biomechanics of Articular Cartilage and Meniscus,” Matrix Biology 85–86 (2020): 47–67, 10.1016/j.matbio.2019.10.001. PMC7137252 31655293

[35] A. L. Fidler , S. P. Boudko , A. Rokas , and B. G. Hudson , “The Triple Helix of Collagens—An Ancient Protein Structure That Enabled Animal Multicellularity and Tissue Evolution,” Journal of Cell Science 131, no. 7 (2018): jcs203950, 10.1242/jcs.203950. PMC5963836 29632050

[36] R. E. Wilusz , J. Sanchez‐Adams , and F. Guilak , “The Structure and Function of the Pericellular Matrix of Articular Cartilage,” Matrix Biology 39 (2014): 25–32, 10.1016/j.matbio.2014.08.009. PMC4198577 25172825

[37] A. D. Theocharis , S. S. Skandalis , C. Gialeli , and N. K. Karamanos , “Extracellular Matrix Structure,” Advanced Drug Delivery Reviews 97 (2016): 4–27, 10.1016/j.addr.2015.11.001. 26562801

[38] C. M. Kielty , S. P. Whittaker , M. E. Grant , and C. A. Shuttleworth , “Type VI Collagen Microfibrils: Evidence for a Structural Association With Hyaluronan,” Journal of cell Biology 118 (1992): 979–990, 10.1083/jcb.118.4.979. PMC2289577 1323568

[39] A. Chevrier , M. Nelea , M. B. Hurtig , C. D. Hoemann , and M. D. Buschmann , “Meniscus Structure in Human, Sheep, and Rabbit for Animal Models of Meniscus Repair,” Journal of Orthopaedic Research 27 (2009): 1197–1203, 10.1002/jor.20869. 19242978

[40] K. Fei , B. D. Andress , A. M. Kelly , D. A. D. Chasse , and A. L. McNulty , “Meniscus Gene Expression Profiling of Inner and Outer Zone Meniscus Tissue Compared to Cartilage and Passaged Monolayer Meniscus Cells,” Scientific Reports 14 (2024): 27423–27423, 10.1038/s41598-024-78580-3. PMC11550462 39521910

[41] B. Cillero‐Pastor , G. B. Eijkel , A. Kiss , F. J. Blanco , and R. M. A. Heeren , “Matrix‐Assisted Laser Desorption Ionization–Imaging Mass Spectrometry: A new Methodology to Study Human Osteoarthritic Cartilage,” Arthritis & Rheumatism 65 (2013): 710–720, 10.1002/art.37799. 23280504

[42] M. Winter , A. Tholey , S. Krüger , H. Schmidt , and C. Röcken , “MALDI‐Mass Spectrometry Imaging Identifies Vitronectin as a Common Constituent of Amyloid Deposits,” Journal of Histochemistry & Cytochemistry 63 (2015): 772–779, 10.1369/0022155415595264. PMC4823803 26101327

[43] I. Schvartz , D. Seger , and S. Shaltiel , “Vitronectin,” International Journal of Biochemistry & Cell Biology 31 (1998): 539–544, 10.1016/S1357-2725(99)00005-9. 10399314

[44] K. Date , H. Sakagami , and K. Yura , “Regulatory Properties of Vitronectin and its Glycosylation in Collagen Fibril Formation and Collagen‐Degrading Enzyme Cathepsin K Activity,” Scientific Reports 11 (2021): 12023, 10.1038/s41598-021-91353-6. PMC8187593 34103584

[45] G. Zhang , Y. Ezura , I. Chervoneva , et al., “Decorin Regulates Assembly of Collagen Fibrils and Acquisition of Biomechanical Properties During Tendon Development,” Journal of Cellular Biochemistry 98 (2006): 1436–1449, 10.1002/jcb.20776. 16518859

[46] J. Sanchez‐Adams , R. E. Wilusz , and F. Guilak , “Atomic Force Microscopy Reveals Regional Variations in the Micromechanical Properties of the Pericellular and Extracellular Matrices of the Meniscus,” Journal of Orthopaedic Research 31 (2013): 1218–1225, 10.1002/jor.22362. PMC4037160 23568545

[47] J. Kwok , S. Grogan , B. Meckes , F. Arce , R. Lal , and D. D'Lima , “Atomic Force Microscopy Reveals Age‐Dependent Changes in Nanomechanical Properties of the Extracellular Matrix of Native Human Menisci: Implications for Joint Degeneration and Osteoarthritis,” Nanomedicine: Nanotechnology, Biology and Medicine 10 (2014): 1777–1785, 10.1016/j.nano.2014.06.010. PMC4374607 24972006

[48] Q. Li , F. Qu , B. Han , et al., “Micromechanical Anisotropy and Heterogeneity of the Meniscus Extracellular Matrix,” Acta Biomaterialia 54 (2017): 356–366, 10.1016/j.actbio.2017.02.043. PMC5413404 28242455

[49] V. A. Solarte David , V. R. Güiza‐Argüello , M. L. Arango‐Rodríguez , C. L. Sossa , and S. M. Becerra‐Bayona , “Decellularized Tissues for Wound Healing: Towards Closing the gap Between Scaffold Design and Effective Extracellular Matrix Remodeling,” Frontiers in Bioengineering and Biotechnology 10 (2022): 821852, 10.3389/fbioe.2022.821852. PMC8896438 35252131

[50] E. J. Vanderploeg , C. G. Wilson , S. M. Imler , C. H. Y. Ling , and M. E. Levenston , “Regional Variations in the Distribution and Colocalization of Extracellular Matrix Proteins in the Juvenile Bovine Meniscus,” Journal of Anatomy 221 (2012): 174–186, 10.1111/j.1469-7580.2012.01523.x. PMC3406364 22703476

[51] E. Folkesson , A. Turkiewicz , M. Rydén , et al., “Proteomic Characterization of the Normal Human Medial Meniscus Body Using Data‐Independent Acquisition Mass Spectrometry,” Journal of Orthopaedic Research 38 (2020): 1735–1745, 10.1002/jor.24602. PMC7610686 31989678

[52] S. Chen and D. E. Birk , “The Regulatory Roles of Small Leucine‐Rich Proteoglycans in Extracellular Matrix Assembly,” FEBS Journal 280 (2013): 2120–2137, 10.1111/febs.12136. PMC3651807 23331954

[53] D. Q. Nesbitt , X. Pu , M. W. Turner , et al., “Age‐Dependent Changes in Collagen Crosslinks Reduce the Mechanical Toughness of Human Meniscus,” Journal of Orthopaedic Research 42 (2024): 1870–1879, 10.1002/jor.25824. PMC13073472 38491967

[54] L. Kain , O. G. Andriotis , P. Gruber , et al., “Calibration of Colloidal Probes With Atomic Force Microscopy for Micromechanical Assessment,” Journal of the Mechanical Behavior of Biomedical Materials 85 (2018): 225–236, 10.1016/j.jmbbm.2018.05.026. 29933150

[55] S. V. Kontomaris , A. Malamou , and A. Stylianou , “The Hertzian Theory in AFM Nanoindentation Experiments Regarding Biological Samples: Overcoming Limitations in Data Processing,” Micron 155 (2022): 103228, 10.1016/j.micron.2022.103228. 35124406

[56] M. Rufin , M. Nalbach , M. Rakuš , et al., “Methylglyoxal Alters Collagen Fibril Nanostiffness and Surface Potential,” Acta Biomaterialia 189 (2024): 208–216, 10.1016/j.actbio.2024.08.039. 39218277

