# Supplementary material for: Spatial Characterization of the Human Meniscus: Bridging Biomechanics and Biochemistry Using Atomic Force Microscopy Micro‐Indentation and Mass Spectrometry Imaging
Source: Small Methods. 2026 Aug 11;10(18):e70908. doi: 10.1002/smtd.70908 (PMC13599714; doi:10.1002/smtd.70908)
Supplement: Supplementary file 1 [file SMTD-10-e70908-s001.docx]

**Supporting Informatio****n**

**Spatial Characterization of the Human Meniscus: Bridging Biomechanics and Biochemistry Using Atomic Force Microscopy Micro-indentation and Mass Spectrometry Imaging**

*Aleksandra N. Lebedeva(a), Martin Handelshauser(a), Orestis G. Andriotis(a), Lena Hirtler(c), Martina Marchetti-Deschmann*(b) and Philipp J. Thurner*(a)*

a) A. N. Lebedeva, M. Handelshauser, Dr. O. G. Andriotis, Univ.Prof. Dr. P. J. Thurner

TU Wien, Faculty of Mechanical and Industrial Engineering

Institute of Lightweight Design and Structural Biomechanics

Gumpendorfer Straße 7, 1060, Vienna Austria

E-mail: [philipp.thurner@tuwien.ac.at](mailto:philipp.thurner@tuwien.ac.at)

b) Univ.Prof. Dr. M. Marchetti-Deschmann

TU Wien, Faculty of Technical Chemistry

Institute of Chemical Technology and Analytics

Getreidemarkt 9, 1060, Vienna Austria

E-mail: [martina.marchetti-deschmann@tuwien.ac.at](mailto:martina.marchetti-deschmann@tuwien.ac.at)

c) Prof. Dr. L. Hirtler

Center for Anatomy and Cell Biology

Medical University of Vienna

Schwarzspanierstraße 17, 1090 Vienna Austria

**Experiment 1**

**Figure S1.** Representative MALDI-FTICR mass spectrum of the first meniscus section after on-tissue digestion with Trypsin/Lys-C.

**Figure S2.** Duplicate of Fig.2

**Figure S3.** Analysis of the first tissue section. a) An optical image of a human meniscus section after MALDI MSI analysis with marked AFM indented regions. Ion images with a spatial distribution of the peptides normalized to TIC: b) m/z 779.4060, clusterin (CLU), unmodified, c) m/z 1076.5201, clusterin (CLU), deamidated (NQ), d) m/z 954.5128, prolargin (PRELP), unmodified, e) m/z 1070.5965, prolargin (PRELP), unmodified, f) m/z 1314.6797, vitronectin (VTNC), unmodified, g) m/z 1422.6502, vitronectin (VTNC), unmodified, h) m/z 1176.5440, decorin (PGS2), unmodified, i) m/z 1325.5835, decorin (PGS2), unmodified.

**Figure S4.** Duplicate of Fig.S3

**Experiment 2**

**Figure S5.** Representative MALDI-FTICR mass spectrum of the second meniscus section after on-tissue digestion with Trypsin/Lys-C.

**Figure S6.** Analysis of the second tissue section. a) An optical image of a human meniscus section after MALDI MSI analysis with marked AFM indented regions. Ion images with a spatial distribution of the peptides normalized to TIC: b) m/z 836.4376, collagen type I alpha 1 chain/collagen type II alpha 1 chain/collagen type III alpha 1 chain (COL1α1/ COL2α1/COL3α1), unmodified, c) m/z 868.4263, collagen type I alpha 1 chain/collagen type II alpha 1 chain (COL1α1/ COL2α1), unmodified, d) m/z 1094.5944, collagen type III alpha 1 chain (COL3α1), 2 P-oxidation (ox), e) m/z 1095.5666, collagen type I alpha 2 chain (COL1α2), unmodified, f) m/z 1297.6111, collagen type I alpha 1 and alpha 2 chains (COL1α1/α2), unmodified, g) m/z 1138.5657, collagen type II alpha 1 chain (COL2α1), acetylation (ac), h) m/z 1267.6725, collagen type VI alpha 3 chain (COL6α3), unmodified, i) m/z 1774.8070, collagen type VI alpha 3 chain (COL6α3), deamidation (de).

**Figure S7.** Duplicate of Fig.S6.

**Figure S8.** Analysis of the second tissue section. a) An optical image of a human meniscus section after MALDI MSI analysis with marked AFM indented regions. Ion images with a spatial distribution of the peptides normalized to TIC: b) m/z 779.4060, clusterin (CLU), unmodified, c) m/z 1076.5201, clusterin (CLU), deamidated (NQ), d) m/z 954.5128, prolargin (PRELP), unmodified, e) m/z 1070.5965, prolargin (PRELP), unmodified, f) m/z 1314.6797, vitronectin (VTNC), unmodified, g) m/z 1422.6502, vitronectin (VTNC), unmodified, h) m/z 1176.5440, decorin (PGS2), unmodified, i) m/z 1325.5835, decorin (PGS2), unmodified. Scale bar: 2 mm.

**Figure S9.** Duplicate of Fig.S8.

**Figure S10.** Analysis of the second tissue section. a) An optical image of a peripheral human meniscus section after MALDI MSI analysis with marked AFM indented regions; b) MALDI MSI segmentation (bisecting k-means clustering) of a human meniscus section; c) Corresponding dendrogram illustrating the hierarchical relationship of the identified clusters. Indentation modulus maps of the marked AFM indented regions: d) red-red (outer), e) red-white (middle), f) white-white (inner). Created with BioRender.com.

**Figure S11.** Analysis of the second section. Heatmap of TIC-normalized peptide ion intensities normalized per m/z across the three meniscal zones (RR, RW, WW), with the maximum regional mean intensity for each m/z set to 100%. Mean indentation modulus values are shown. *Unique peptides; **m/z 1297.6111 – collagen I peptide (α1/α2).

**Experiment 3**

**Figure S12.** Representative MALDI-FTICR mass spectrum of the third meniscus section after on-tissue digestion with Trypsin/Lys-C.

**Figure S13.** Analysis of third tissue section. a) An optical image of a peripheral human meniscus section after MALDI MSI analysis with marked AFM indented regions. Ion images with a spatial distribution of the peptides normalized to TIC: b) m/z 836.4376, collagen type I alpha 1 chain/collagen type II alpha 1 chain/collagen type III alpha 1 chain (COL1α1/ COL2α1/COL3α1), unmodified, c) m/z 868.4263, collagen type I alpha 1 chain/collagen type II alpha 1 chain (COL1α1/ COL2α1), unmodified, d) m/z 1094.5944, collagen type III alpha 1 chain (COL3α1), 2 P-oxidation (ox), e) m/z 1095.5666, collagen type I alpha 2 chain (COL1α2), unmodified, f) m/z 1297.6111, collagen type I alpha 1 and alpha 2 chains (COL1α1/α2), unmodified, g) m/z 1138.5657, collagen type II alpha 1 chain (COL2α1), acetylation (ac), h) m/z 1267.6725, collagen type VI alpha 3 chain (COL6α3), unmodified, i) m/z 1774.8070, collagen type VI alpha 3 chain (COL6α3), deamidation (de).

**Figure S14.** Duplicate of Fig.S13.

**Figure S15.** Analysis of third tissue section. a) An optical image of a peripheral human meniscus section after MALDI MSI analysis with marked AFM indented regions. Ion images with a spatial distribution of the peptides normalized to TIC: b) m/z 779.4060, clusterin (CLU), unmodified, c) m/z 1076.5201, clusterin (CLU), deamidated (NQ), d) m/z 954.5128, prolargin (PRELP), unmodified, e) m/z 1070.5965, prolargin (PRELP), unmodified, f) m/z 1314.6797, vitronectin (VTNC), unmodified, g) m/z 1422.6502, vitronectin (VTNC), unmodified, h) m/z 1176.5440, decorin (PGS2), unmodified, i) m/z 1325.5835, decorin (PGS2), unmodified.**Figure S16.** Duplicate of Fig. S15.

**Figure S17.** Analysis of third tissue section. a) An optical image of a peripheral human meniscus section after MALDI MSI analysis with marked AFM indented regions; b) MALDI MSI segmentation (bisecting k-means clustering) of a human meniscus section; c) Corresponding dendrogram illustrating the hierarchical relationship of the identified clusters. Indentation modulus maps of the marked AFM indented regions: d) red-red (outer), e) red-white (middle), f) white-white (inner). Created with BioRender.com.

**Figure S18.** Analysis of third tissue section. Heatmap of TIC-normalized peptide ion intensities normalized per m/z across the three meniscal zones (RR, RW, WW), with the maximum regional mean intensity for each m/z set to 100%. Mean indentation modulus values are shown. *Unique peptides; **m/z 1297.6111 – collagen I peptide (α1/α2).

**Figure S19.** Sample preparation: Tragacanth mixture in pyramidal shape (a); Embedded part of the meniscus in tragacanth (b).

**Figure S20.** Examples of the force-indentation curves: approach (blue) and retraction (red).

**Figure S21.** Approach curves (blue) from a single force map 10 x 10 um (8 x 8 pixels).

**Figure S22.** Distribution of mean indentation modulus values across ROIs within the three meniscal zones (RR, RW, and WW) in the first (a), second (b), and third (c) meniscal sections.
